# Endocrine society 2025 diagnostic criteria increase primary aldosteronism detection in hypertensive patients: a comparative study with 2016 guidelines

**DOI:** 10.1016/j.ijcrp.2026.200638

**Published:** 2026-04-12

**Authors:** Clara Cherdo, Corina Mirea, Camille Zamperini, Solène Brouder, Dominique Stephan, Elena-Mihaela Cordeanu

**Affiliations:** a Department of Hypertension and Vascular Diseases, Clinical Pharmacology, Strasbourg University Hospitals, Strasbourg, France; b Translational Cardiovascular Medicine, UR 3074, CRBS, University of Strasbourg, Strasbourg, France

**Keywords:** Primary aldosteronism, Secondary hypertension, Aldosterone-to-renin ratio, Endocrine society guidelines, Diagnostic criteria

## Abstract

**Background:**

Primary aldosteronism (PA) is the most common cause of secondary hypertension, associated with disproportionate cardiovascular morbidity. The 2025 Endocrine Society (ES) guidelines revised diagnostic criteria, allowing diagnosis based on a biochemical triad (suppressed renin, elevated aldosterone and aldosterone-to-renin ratio, ARR) without mandatory confirmatory testing. We compared PA detection and diagnostic performance using ES 2016 versus ES 2025 algorithms in hypertensive patients referred to a specialized center.

**Methods:**

We retrospectively analyzed 137 consecutive patients referred for secondary hypertension work-up to Strasbourg University Hospital (June 2024 - June 2025). All underwent standardized hormonal evaluation and saline infusion testing (SIT). PA was defined according to ES 2016 (ARR >23 ng/mUI with aldosterone ≥200 ng/L, or 90–200 ng/L with positive saline test) and ES 2025 criteria with locally adapted thresholds (ARR >18.7 ng/ng, renin ≤6.15 ng/L, aldosterone ≥75 ng/L).

**Results:**

PA prevalence increased from 8.8% (n = 12) with ES 2016 to 16.1% (n = 22) with ES 2025 criteria (p = 0.009). Diagnostic concordance was high (91.7%, κ = 0.61). Net reclassification improvement ranged from +148% to +175%. Newly identified patients exhibited milder PA phenotypes with less severe/resistant hypertension (27% vs 73%, p = 0.03), lower aldosterone levels (95 vs 185 ng/L, p < 0.001), and predominantly negative SIT (82%). Despite a 50% false-negative rate, positive SIT remained the only independent predictor of PA diagnosis (OR = 3.25, 95%CI 1.22-8.67, p = 0.019).

**Conclusion:**

ES 2025 criteria increase PA detection by identifying milder forms not captured by previous algorithms. These findings support a phenotype-based diagnostic approach and question the systematic use of confirmatory testing.

## Highlights

• ES 2025 criteria nearly doubled PA detection compared with ES 2016.
• Diagnostic gain reflects a shift in case definition rather than ARR threshold changes.
• Newly identified patients show milder phenotypes with preserved aldosterone suppressibility.
• High false-negative rate of SIT supports reduced reliance on confirmatory testing.

## 1 Introduction

Primary aldosteronism (PA) is recognized as the most frequent and yet underdiagnosed cause of secondary hypertension (HTN), with a global pooled prevalence of 9.4% (95%CI 8.3-10.5%) among unselected hypertensive individuals and up to 29% in resistant forms [[1], [2], [3]]. Compared with essential HTN matched for blood pressure (BP), PA carries disproportionate cardiovascular morbidity including 2-to-4-fold increased risks of stroke, myocardial infarction, atrial fibrillation, and heart failure [[4], [5], [6]]. Mendelian randomization studies have strengthened the evidence for causal, aldosterone-mediated cardiovascular harm independent of BP elevation, and for bidirectional interactions between PA and metabolic disorders including type 2 diabetes and obesity [7,8].

Historically considered a rare condition characterized by HTN, hypokalemia, and adrenal adenoma, PA is now understood as a continuum of autonomous aldosterone secretion [[9], [10], [11]]. Even subclinical forms are associated with arterial stiffness and adverse cardiac remodeling, independently of BP [12,13]. Early identification enables specific treatment, surgical adrenalectomy for unilateral disease (35% of cases) or mineralocorticoid receptor antagonists (MRAs) for bilateral forms (65% of cases), reducing morbidity and mortality [14]. Yet traditional diagnostic strategies relying on restrictive screening and mandatory confirmatory testing have systematically missed milder or atypical presentations. Until recently, the 2016 Endocrine Society (ES 2016) clinical practice guidelines on PA, recommended selective screening for PA in patients with severe or resistant HTN, hypokalemia, or adrenal incidentalomas [1]. The updated Endocrine Society 2025 guidelines (ES 2025) have redefined the diagnostic landscape by advocating systematic biochemical screening of all hypertensive patients (conditional recommendation, low-quality evidence) and establishing simplified thresholds for aldosterone, renin, and the aldosterone-to-renin ratio (ARR) [15]. A recent Italian survey revealed PA screening remains predominantly restricted to resistant HTN, with systematic screening occurring in fewer than one-third of centers and only two PA cases diagnosed annually per center [16]. Accumulating evidence has questioned the necessity and reliability of confirmatory tests, particularly the saline infusion test (SIT), which demonstrates substantial false-negative rates [10,[17], [18], [19], [20]]. By abandoning confirmatory testing for high-probability cases and adopting a biological phenotype-driven approach, the 2025 recommendations aim to capture earlier stages of the disease [15]. However, real-world evidence comparing these diagnostic approaches remains limited, and a gap persists between expected prevalence and actual detection [21]. The present study aimed to evaluate how algorithm choice affects diagnostic yield in patients who do reach biochemical screening, by directly comparing diagnostic agreement, prevalence, and clinical profiles between ES 2016 and ES 2025 criteria in consecutive hypertensive patients referred to a specialized HTN center, labeled European "Blood Pressure Clinic" [22].

## 2 Material and methods

### 2.1 Study population

We conducted a retrospective analysis of consecutive patients referred to the HTN Day Hospital at Strasbourg University Hospital between June 2024 and June 2025 for secondary HTN work-up by community-based cardiologists. Referral indications thus included, but were not limited to, classical high-risk features; patients were referred based on clinical judgment of the referring physician. Within this referred population, biochemical PA screening was performed systematically in all patients at our centre. Inclusion criteria required availability of simultaneous aldosterone and direct renin concentration (DRC) measurements. Exclusion criteria included missing ARR measurements due to technical issues and duplicate hospitalizations during the study period. The study protocol was approved by the institutional ethics committee (registration R25-038) and complied with the Declaration of Helsinki.

### 2.2 Clinical and hormonal assessment

All patients underwent standardized clinical evaluation including detailed medical history, physical examination, and laboratory testing. Cardiovascular risk stratification included evaluation of traditional risk factors, SCORE 2 risk stratification and assessment for established cardiovascular disease.

Hormone measurements were obtained under standardized conditions with morning collection after at least 2 h upright posture followed by 15 min seated rest. Plasma aldosterone concentration (PAC) and direct renin concentration (DRC) were measured using automated chemiluminescent immunoassay (iSYS, IDS Corporation). Following established recommendations for laboratory-specific threshold validation [[23], [24], [25]], our institution performed inter-laboratory comparison studies demonstrating that our iSYS platform (IDS Corporation) yields DRC values approximately 25% higher than French reference laboratories using alternative immunoassay methods (Supplementary Methods). Thresholds were derived accordingly: ARR >18.7 ng/ng (PAC ng/L / DRC ng/L), DRC ≤6.15 ng/L and PAC ≥75 ng/L represent the locally validated equivalent thresholds of ES 2025 entry criteria for PA. The ES 2016 local threshold of ARR >23 ng/mIU (PAC ng/L / DRC mIU/L) was validated specifically for the iSYS platform [26] and is consistent with the ES 2016 guideline range of accepted cutoffs (equivalent range: 24-49 when PAC is expressed in ng/L and DRC in mIU/L, corresponding to the guideline's 38-77 ng/ng). The full unit-standardized comparison between ES 2016 and ES 2025 ARR thresholds across all measurement systems is provided in Table 1.

**Table 1:** Comparison of diagnostic thresholds: ES 2016, ES 2025, and locally adapted criteria.

Parameter	ES 2016	ES 2025	Our study (local threshold)	Rationale for local adaptation
***Screening and diagnostic criteria***				
**Target population**	Severe/resistant HTN, hypokalemia, adrenal incidentaloma	All hypertensive patients	All hypertensive patients referred for secondary HTN work-up in a tertiary center	Aligned with ES 2025
**ARR threshold**	**> 23** (ng/mUI) > **64** (pmol/mUI) **> 38** (ng/ng)	*Immunoassay:* **>70** (pmol/mUI) > **42.1** (ng/ng) *LC-MS/MS:* **>52** (pmol/mUI) > **31.2** (ng/ng)	**>18.7 (ng/ng)**	iSYS-specific validation (Manolopoulou et al., 2015, n = 152): 99% sensitivity, 79% specificity. iSYS aldosterone correlates with LC-MS/MS (3.2% difference); threshold aligns with LC-MS/MS-based cutoffs. 25% higher DRC on iSYS mechanistically reduces ARR
**Suppressed renin (DRC)**	**≤3 ng/L** (technical sensitivity threshold)	**≤8.2 mU/L** (4.92 ng/L)	**≤6.15 ng/L**	8.2 mU/L × 1.25 (iSYS overestimation) = 10.25 mU/L; converted: 10.25 ÷ 1.667 = **6.15 ng/L**
**Elevated aldosterone (PAC)**	**≥200 ng/L** (certain PA) **90**–**200 ng/L** (probable; requires SIT) **<90 ng/L** (PA excluded)	*Immunoassay:* **≥100 ng/L** (277 pmol/L) *LC-MS/MS:* **≥75 ng/L** (208 pmol/L)	**≥75 ng/L**	Local iSYS aldosterone correlates with LC-MS/MS (mean difference 3.2%); LC-MS/MS threshold applies: ≥75 ng/L
**Confirmatory test**	Required if ARR >23ng/mUI and PAC 90–200 ng/L	Not required for high-probability; shared decision-making for intermediate probability	SIT performed systematically as for local practices	Aligned with ES 2025
**Post-SIT aldosterone (permissive)**	**>50 ng/L**	**>50 ng/L**	**>50 ng/L**	No adjustment needed
**Post-SIT aldosterone (restrictive)**	**>100 ng/L**	**>100 ng/L**	**>100 ng/L**	No adjustment needed
***Lateralization probability assessment (ES 2025 management algorithm)***				
**High lateralization probability**	Not specifically definedAC	**Hypokalemia** + **DRC <2 mU/L***(Immunoassay/LC-MS/MS)* + **PAC >200 ng/L** (Immunoasssay)/PAC >150 ng/L (LC-MS/MS)	**Hypokalemia + DRC <1.5 ng/L + PAC >150 ng/L**	DRC: 2 mU/L × 1.25 = 2.5 mU/L ÷ 1.667 = **1.5 ng/L**. PAC: Local iSYS correlates with LC-MS/MS; LC-MS/MS threshold applies (>150 ng/L)
**Low lateralization probability**	Not specifically defined	**Normokalemia** + **PAC <110 ng/L** (*Immunoassay*)/PAC <80 ng/L (*LC-MS/MS*)	**Normokalemia + PAC <80 ng/L**	Local iSYS correlates with LC-MS/MS; LC-MS/MS threshold applies (<80 ng/L). No PA patient in our cohort met this criterion^a^
**Intermediate lateralization probability**	Not specifically defined	All PA patients not meeting high- or low-probability criteria	All PA patients not meeting high- or low-probability criteria	Aligned with ES 2025

ARR: aldosterone-to-renin ratio; DRC: direct renin concentration; HTN: hypertension; iSYS: IDS automated chemiluminescent immunoassay platform; LC-MS/M: liquid chromatography–tandem mass spectrometry; PA: primary aldosteronism; PAC: plasma aldosterone concentration; SIT: saline infusion test. Unit conversions: mU/L to ng/L: ÷1.667; ng/L to ng/dL: ÷10; ng/L to pmol/L: × 2.774.

∗ES 2016 ARR threshold of >23 (ng/mUI) used DRC in mUI/L; equivalent values in other units depend on assay platform.

a Low lateralization probability requires normokalemia with PAC <80 ng/L. Since PA diagnosis requires PAC ≥75 ng/L, only patients with PAC 75–80 ng/L and normokalemia could theoretically qualify; no patient in our cohort met these criteria.

### 2.3 Saline infusion test

All patients underwent a standardized SIT with 2 L of 0.9% NaCl infused over 4 h, as part of our institutional protocol for phenotypic characterization and lateralization probability assessment. Hormones were measured before and after infusion. Aldosterone >50 ng/L after infusion indicated incomplete suppression (“permissive” threshold), whereas >100 ng/L indicated definitive non-suppression (“restrictive” threshold). Both thresholds were analyzed for diagnostic performance. Under ES 2025 criteria, PA diagnosis was established based solely on the baseline biochemical triad (suppressed renin + elevated aldosterone + elevated ARR), independently of SIT result [15].

### 2.4 Lateralization probability assessment

Lateralization probability was classified according to the ES 2025 algorithm which stratifies PA patients based on biochemical severity [15]. "High probability" of lateralizing PA was defined by biochemical features of severe PA: hypokalemia combined with very low renin (DRC <2 mIU/L per guidelines, corresponding to <1.5 ng/L at our institution) and elevated aldosterone (>15 ng/dL LC-MS/MS as per guidelines corresponding to the same value at our institution). "Low probability" was defined as normokalemia with aldosterone <80 ng/L, for which pursuing aldosterone suppression testing is not considered necessary. "Intermediate probability" included all other PA patients not meeting high- or low-probability criteria, for whom the guidelines recommend shared decision-making regarding an empiric trial of MRA versus proceeding to aldosterone suppression testing ± adrenal venous sampling (AVS), based on surgical candidacy and patient preference [15].

### 2.5 Statistical analysis

Continuous variables were expressed as median [interquartile range] or mean ± SD, as appropriate and frequencies with percentages for categorical variables. Comparisons between groups used Mann-Whitney *U* test or Kruskal-Wallis for continuous variables and Chi-square or Fisher's exact test for categorical variables. Sensitivity analyses were performed to assess the potential impact of missing data. Best-case and worst-case scenarios assumed all excluded patients were PA-positive or PA-negative, respectively. Baseline characteristics of excluded versus included patients were compared to assess potential selection bias. Diagnostic performance evaluation using ES 2025 algorithm as reference, calculating sensitivity, specificity, positive and negative predictive values (PPV, NPV) with exact binomial confidence intervals (CI). Agreement between diagnostic strategies was assessed using Cohen's kappa coefficient and net reclassification improvement (NRI) quantified diagnostic enhancement provided by ES 2025 criteria. Multivariate logistic regression identified independent predictors of PA diagnosis. Variables demonstrating univariate associations (p < 0.20) and clinical relevance were incorporated into the predictive model. Model quality was verified by multicollinearity assessment and calibration analysis. All tests were two-sided, with p < 0.05 considered significant. Analyses were performed using R software (version 4.4.1).

## 3 Results

### 3.1 Patient characteristics

**Fig. 1:**
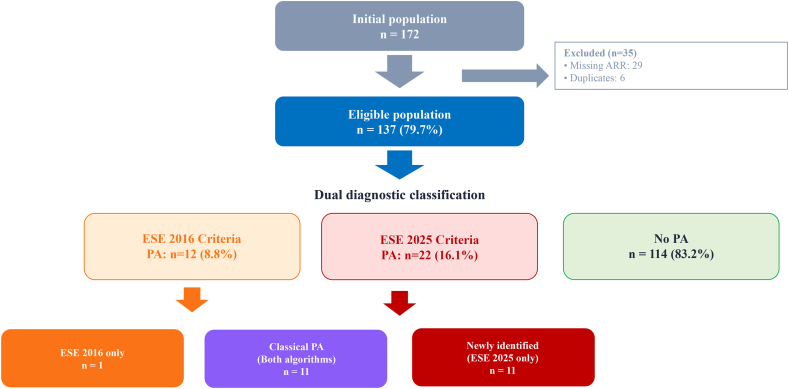
**Study flowchart illustrating patient selection and diagnostic classification of primary aldosteronism according to Endocrine Society 2016 and 2025 criteria.** ARR**:** aldosterone-to-renin ratio; ES: Endocrine Society guidelines; PA**:** primary aldosteronism; SIT**:** saline infusion test.

Among the 172 patients consecutively admitted for etiological work-up between June 2024 and June 2025, 137 met inclusion criteria after excluding 29 patients with missing hormonal data and 6 duplicates (Fig. 1). The cohort included 73 men (53.3%) with a median age of 41 years (IQR 34-53). Nearly half of the patients (48.2%) had recent-onset HTN (<1 year), while 39.4% presented with severe HTN and 19.7% with resistant HTN. Before interfering medications wash-out, the mean number of antihypertensive drugs was 2.1 ± 1.3, most frequently calcium channel blockers (62.8%), renin angiotensin system (RAS) inhibitors (53.3%), and diuretics (22.6%). MRAs were used in 6.6%. Comorbid conditions included dyslipidemia in 62% of patients, obesity (BMI ≥30 kg/m^2^) in 26%, current smoking in 14%, and diabetes in 6%. The mean SCORE2 cardiovascular risk was 4.2 ± 3.8%. Established cardiovascular disease was present in 6% (ischemic heart disease), 3% (atrial fibrillation), and 3% (prior ischemic stroke). Left ventricular hypertrophy was documented in 11% and median eGFR was 104 mL/min/1.73 m^2^ (IQR 90-115) (Table 2). Hormonal results are presented in Supplementary Table S1. Comparison of excluded (n = 29) versus included patients revealed no significant differences except for age (median 52 vs 41 years, p < 0.01) (Supplementary Table S2). Sensitivity analysis demonstrated that prevalence estimates remained robust: best-case scenario (all excluded PA-positive) yielded 30.7% prevalence, while worst-case scenario (all excluded PA-negative) maintained 13.3% prevalence with ES 2025 criteria (Supplementary Table S3).

**Table 2:** Baseline characteristics of the study population.

Characteristic	Total (n = 137)	ES 2016 (n = 12)	ES 2025 (n = 22)	No PA (n = 114)	p-value∗
**Demographics**					
Age, years, median [IQR]	41 [34-53]	47.5 [43-64]	44.5 [38-59]	41 [33-52]	0.28
Male sex, n (%)	73 (53.3)	8 (66.7)	13 (59.1)	60 (52.6)	0.58
BMI, kg/m^2^, mean ± SD	27.8 ± 5.2	29.2 ± 4.8	28.1 ± 5.5	27.6 ± 5.2	0.71
**Hypertension characteristics**					
Recent onset (<1 year), n (%)	66 (48.2)	6 (50.0)	12 (54.5)	54 (47.4)	0.55
HTA duration, years, mean ± SD	5.2 ± 8.1	4.8 ± 6.2	6.1 ± 9.8	5 ± 7.9	0.63
Severe hypertension, n (%)	54 (39.4)	9 (75.0)	9 (40.9)	44 (38.6)	0.84
Resistant hypertension, n (%)	27 (19.7)	5 (41.7)	7 (31.8)	20 (17.5)	0.15
Office SBP, mmHg, median [IQR]	150 [136-169]	162.5 [147-183]	148.5 [140-177]	150 [136-167]	0.74
Office DBP, mmHg, median [IQR]	89 [80-97]	89.5 [86-100]	88.5 [81-98]	89 [79-97]	0.89
**Antihypertensive therapy**					
Number of antihypertensive drugs, mean ± SD	2.1 ± 1.3	2.4 ± 1.1	2.3 ± 1.4	2.0 ± 1.3	0.39
RAS inhibitors, n (%)	73 (53.3)	5 (41.7)	10 (45.5)	63 (55.3)	0.42
Calcium blockers, n (%)	86 (62.8)	11 (91.7)	17 (77.3)	68 (59.6)	0.13
Diuretics, n (%)	31 (22.6)	3 (25.0)	4 (18.2)	27 (23.7)	0.58
Betablockers - n (%)	29 (21.2)	4 (33.3)	5 (22.7)	24 (21.1)	0.87
MRAs, n (%)	9 (6.6)	1 (8.3)	3 (13.6)	6 (5.3)	0.20
Good treatment adherence^a^, n (%)	85/120 (70.8)	8/10 (80.0)	16/19 (84.2)	65/95 (68.4)	0.18
**Cardiovascular risk factors**					
Dyslipidemia, n (%)	78/125 (62.4)	8/12 (66.7)	14/22 (63.6)	62/105 (59.0)	0.69
Obesity (BMI ≥30), n (%)	32/121 (26.4)	4/11 (36.4)	6/21 (28.6)	24/95 (25.3)	0.77
Smoking, n (%)	45/125 (36)	5/12 (41.7)	8/21 (38.1)	35/105 (33.3)	0.68
Diabetes, n (%)	8/136 (5.9)	1/12 (8.3)	2/22 (9.1)	6/114 (5.3)	0.69
SCORE2, %, mean ± SD	4.2 ± 3.8	6.8 ± 4.5	5.1 ± 4.2	3.8 ± 3.2	0.25
**Comorbidities**					
Stroke, n (%)	4/135 (3)	1/12 (8.3)	1/22 (4.5)	3/114 (2.6)	0.68
Ischemic heart disease, n (%)	8/135 (5.9)	1/12 (8.3)	1/22 (4.5)	7/114 (6.1)	0.79
Atrial fibrillation, n (%)	4/134 (3)	1/12 (8.3)	0/22	3/113 (2.6)	0.59
**Laboratory findings**					
Hypokalemia (≤3.5 mmol/L), n (%)	27 (19.7)	5 (41.7)^b^	7 (31.8)	20 (17.5)	0.15
eGFR, mL/min/1.73m^2^, median [IQR]	104 [90-115]	102.5 [99-119]	102.5 [90-115]	104 [88-115]	0.82

BMI: body mass index; DBP: diastolic blood pressure; eGFR: estimated glomerular filtration rate; ES: Endocrine Society; IQR: interquartile range; PA: primary aldosteronism; RAS: renin angiotensin system; SBP: systolic blood pressure; SD: standard deviation. ∗p-values represent comparisons between ES 2025 and No PA groups.

a Adherence was defined as good if at least 50% of the prescribed molecules were identified in plasma samples.

b p-value for hypokalemia was 0.04 when comparing ES 2016 vs No PA groups.

### 3.2 PA prevalence and diagnostic agreement between algorithms

Application of the 2025 ES algorithm substantially increased diagnostic yield compared with the 2016 version. Using locally adapted thresholds, 22 patients (16.1%) met 2025 criteria for PA versus 12 (8.8%) with the 2016 algorithm (p = 0.009), representing a 92% relative increase in detection (Supplementary Fig. S1).

Diagnostic concordance between algorithms was 91.7%, with 11 of 12 ES 2016-positive patients also meeting ES 2025 criteria (κ = 0.61, 95%CI 0.42-0.80) (Supplementary Table S4). One patient meeting ES 2016 criteria failed to meet ES 2025 criteria due to marginally elevated renin concentration. Taking ES 2025 criteria as reference, ES 2016 criteria demonstrated excellent specificity (99%) but lower sensitivity (50% permissive, 23% restrictive) (Supplementary Table S5). Net reclassification improvement provided by ES 2025 criteria versus ES 2016 reached +148% (using the SIT permissive threshold) and +175% (using the SIT restrictive threshold) (Supplementary Table S6).

Comparative analyses between patients with PA diagnosed according to ES 2016 and ES 2025 algorithms, and without PA (“No PA”) revealed overlapping demographic characteristics but distinct biochemical profiles. Both PA groups showed markedly suppressed plasma renin (median 2.1 vs 12.2 ng/L in non-PA; p < 0.001) and more frequent hypokalemia (p = 0.04) compared to non-PA patients (Table 2, Supplementary Table S1).

The 11 newly diagnosed patients, defined as “milder PA”, met ES 2025, but not ES 2016 criteria and exhibited distinct clinical and biochemical features compared to classical PA cases: less frequent severe or resistant HTN (27% vs 73%, p = 0.03), lower baseline aldosterone levels (95 [85-125] vs 185 [145-210] ng/L, p < 0.001), lower ARR values (22.1 [[19], [20], [21], [22], [23], [24], [25], [26]] vs 28.5 [[22], [23], [24], [25], [26], [27], [28], [29], [30], [31], [32], [33], [34], [35], [36], [37], [38], [39], [40], [41], [42], [43], [44], [45]], p = 0.04), and predominantly negative SIT at the permissive threshold (82% vs 18%, p = 0.002) (Table 3).

**Table 3:** Characteristics of concordant vs discordant PA cases between the ES 2016 and 2025 diagnostic algorithms.

Characteristic	Classical PA (Both algorithms) (n = 11)	Newly identified PA (ES 2025 only) (n = 11)	p-value
**Clinical characteristics**			
Severe/resistant HTN, n (%)	8 (73)	3 (27)	**0.03∗**
Hypokalemia (≤3.5 mmol/L), n (%)	5 (45)	2 (18)	0.36
Recent onset HTN (<1 year), n (%)	5 (45)	6 (55)	0.67
**Biochemical parameters**			
Baseline aldosterone, ng/L, median [IQR]	185 [145-210]	95 [85-125]	**<0.001∗**
ARR, median [IQR]	28.5 [22-45]	22.1 [19-26]	**0.04∗**
Post-SIT aldosterone, ng/L, median [IQR]	85 [55-110]	42 [38-52]	**<0.001∗**
Negative SIT (≤50 ng/L), n (%)	2 (18)	9 (82)	**0.002∗**
**Lateralization probability**			
High probability, n (%)	5 (45)	1 (9)	0.15
Intermediate probability, n (%)	6 (55)	10 (91)	0.15

ARR: aldosterone-to-renin ratio; ES: Endocrine Society; HTN: hypertension; IQR: interquartile range; PA: primary aldosteronism; SIT: saline infusion test. ∗p < 0.05.

Note: Lateralization probability was classified according to the ES 2025 management algorithm with locally adapted thresholds: high probability = hypokalemia + DRC <1.5 ng/L + aldosterone >150 ng/L; low probability = normokalemia + aldosterone <80 ng/L; intermediate probability = all other PA patients. No patient met low-probability criteria in this cohort. See Methods section 2.4 and Supplementary methods for threshold derivation.

Lateralization probability assessment revealed that 72.7% of all ES 2025-diagnosed patients demonstrated intermediate probability (91% among newly diagnosed patients) compared to 55% of classical cases. High lateralization probability, indicating likely unilateral disease amenable to surgical cure, was present in only 9% of newly diagnosed patients versus 45% of classical cases, suggesting that the majority of newly identified patients will require medical rather than surgical management (Table 3).

### 3.3 Saline infusion test performance

SIT results were available in 125 patients (91%). Using the permissive cut-off (aldosterone >50 ng/L), 40 patients (29.2%) were classified as non-suppressors, while the restrictive cut-off (100 ng/L) identified only 12 (8.8%). When compared to the ES 2025 reference classification, SIT demonstrated moderate diagnostic accuracy, with sensitivity/specificity values of 64%/77% (permissive) and 14%/92% (restrictive), respectively. Conversely, among ES 2025-positive patients, 50% had a negative SIT.

### 3.4 Predictors of PA

Multivariate logistic regression analysis identified factors independently associated with PA diagnosis according to ES 2025 criteria. After adjustment for potential confounding factors, positive saline infusion test emerged as the sole statistically significant independent predictor (adjusted OR = 3.25, p = 0.019). Resistant HTN and hypokalemia maintained similar effect sizes but did not achieve statistical significance in the multivariable model (adjusted OR = 1.95, p = 0.21 and OR = 2.05, p = 0.18, respectively). The model demonstrated acceptable discrimination (AUC = 0.72) and good calibration (Hosmer-Lemeshow p = 0.42), suggesting reasonable predictive performance despite the relatively modest sample size. The events-to-variable ratio of 7.3 met conventional requirements for stable logistic regression modeling (Supplementary Table S7) [27].

## 4 Discussion

Applying the ES 2025 diagnostic criteria nearly doubled PA detection in a real-world HTN referral population, mainly by identifying patients who would previously have been classified as negative. The high inter-algorithm concordance confirms that classical PA phenotypes remain captured, while additional milder forms become detectable. Diagnostic yields may further evolve as clinical practice adapts to the new recommendations and laboratory validation efforts mature across institutions. International data support these findings: the SCREENING-PA study reported 11% prevalence using ARR>70pmol/mU with confirmatory testing (SIT or fludrocortisone), while the CONPASS study found 4-7% prevalence in treatment-naive hypertensive patients using an ARR>55pmol/mIU together with plasma aldosterone >10 ng/dl and confirmatory testing with captopril [28,29]. Notably, both studies relied on confirmatory testing, which, as shown by our data, may underestimate true PA prevalence given the high false-negative rate of confirmatory tests in milder phenotypes. Our locally validated thresholds were derived following pre-existing recommendations for assay-specific calibration, and were subsequently confirmed as concordant with ES 2025 methodology as the study period (June 2024-June 2025) predates the formal publication of ES 2025 guidelines. The increase in detection mainly reflects differences in diagnostic approach. The elimination of mandatory confirmatory testing and the inclusion of the renin suppression criterion appear more influential than threshold adjustments alone, as evidenced by the high negative SIT rate among newly diagnosed patients [19,30]. Patients newly identified by the ES 2025 criteria express a distinct phenotype characterized by aldosterone secretion autonomy with preserved suppressibility reflecting a less advanced stage of the disease. Although aldosterone concentrations were lower than in patients meeting ES 2016 criteria, renin remained suppressed and ARR elevated, fulfilling the biochemical signature of autonomous aldosterone secretion. Per ES 2025, aldosterone suppression testing is suggested only in patients with intermediate lateralization probability who desire and are candidates for surgical adrenalectomy [15]. It is not required in four distinct situations: (1) patients with high-probability biochemical features of severe PA, where false-negative rates are deemed to outweigh false-positive screening risk; (2) patients unwilling or unable to pursue AVS and adrenalectomy, who can be empirically treated with MRA based on screening results alone; (3) patients with known familial hyperaldosteronism germline mutations; and (4) patients with such low lateralization probability that a formal PA diagnosis is not clinically justified.

This severity-dependent performance of SIT reflects aldosterone suppressibility physiology. Patients with more severe PA profiles (identified by ES 2016 criteria) showed 83% SIT positivity, indicating complete autonomous secretion, whereas those with milder disease showed only 18% positivity, preserving volume-mediated suppression capacity despite inappropriate aldosterone production. This pattern is consistent with contemporary models describing PA as a progressive spectrum rather than a binary entity, with gradual loss of physiological regulation over time [11,31]. Thus, SIT may retain value for borderline biochemical profiles, pre-surgical assessment, or precise phenotypic characterization, while being unsuitable as mandatory confirmation [32,33].

Despite these limitations, positive SIT remained the sole independent predictor of ES 2025-defined PA in multivariate analysis. This finding, while seemingly paradoxical, reflects the fact that when SIT is positive, it strongly confirms autonomous secretion, the test's limitation lies in its inability to detect milder disease when negative, not in the specificity of positive results. Additional evidence confirms the limited SIT sensitivity in milder PA phenotypes: Schirpenbach et al. reported 91% sensitivity in hypokalemic PA, but only 57% in normokalemic forms, while Golani et al. showed that shortened 120-min protocols may offer comparable accuracy with improved feasibility [17,20].

The composition of our cohort warrants contextualisation. The 2024 ESC guidelines on HTN, published in August 2024 and therefore in force throughout our entire study period, introduced a Class IIa, Level B recommendation for systematic ARR-based PA screening in all patients with confirmed HTN [34]. This recommendation, by broadening referral indications beyond classical high-risk features such as resistant HTN or hypokalemia, likely contributed to the inclusion of patients with milder biochemical phenotypes in our cohort. This temporal alignment strengthens rather than undermines our findings: the newly identified patients reflect precisely the population that current guidelines now mandate screening, and the ES 2025 diagnostic criteria are specifically designed to capture this milder disease spectrum.

The mechanistic basis for the 11 newly identified patients warrants explicit clarification. Under ES 2016, a positive SIT was required for diagnosis in patients with aldosterone 90-200 ng/L; negative SIT excluded PA diagnosis regardless of the baseline biochemical profile [1]. Under ES 2025, diagnosis rests on the baseline biochemical triad alone; a negative SIT does not override a positive screen in patients who are not surgical candidates. The 82% negative SIT rate in newly identified patients is therefore not a classification inconsistency but the direct mechanistic illustration of the core innovation of ES 2025: milder phenotypes with preserved aldosterone suppressibility are now captured by the biochemical triad before autonomous secretion becomes irreversible.

Furthermore, implementation of ES 2025 guidelines requires systematic laboratory validation. ARR thresholds are highly assay- and unit-dependent, and direct numerical comparison across guidelines without standardization is methodologically invalid [15,[23], [24], [25]]. When expressed in equivalent units, ES 2025 ARR thresholds are equal to or lower than their ES 2016 counterparts across all measurement systems (Table 1), confirming that the increased PA detection observed in our cohort reflects differences in diagnostic strategy, specifically the introduction of a triadic biochemical approach with a lesser role for confirmatory testing, rather than any modification of the ARR numerical threshold itself. Assay-specific threshold adjustment is essential, as exemplified by our 25% elevation in local renin values observed in our laboratory compared to external references [35]. The assay-dependency of ARR thresholds is explicitly acknowledged by the French SFE/SFHTA/AFCE consensus, which states that the plasma renin activity (PRA)-to-DRC conversion factor varies by kit and technique and that a fixed numerical relation between PRA and DRC is probably only theoretic, underscoring that direct numerical comparison of ARR thresholds across platforms without local validation is methodologically inappropriate [36]. Preanalytical protocols are equally critical: specimens require ambient temperature transport to prevent prorenin cryoactivation, which falsely elevates DRC and generates false-negative ARR results [37,38]. At our institution, ES 2025 criteria have been integrated into routine practice through: (1) updated laboratory protocols with method-specific validated thresholds, (2) simplified screening algorithms eliminating confirmatory testing for high-probability cases, (3) renin-guided MRA titration protocols for medical management and (4) AVS referral based on lateralization probability assessment. Emerging therapeutic options, including novel MRAs and aldosterone synthase inhibitors, may help address the increased demand for PA-specific treatment generated by expanded diagnostic criteria [[39], [40], [41]].

Detecting these milder forms carries clinical importance beyond BP control [12]. Even modest aldosterone excess directly damages cardiovascular tissues through mineralocorticoid receptor activation, contributing to vascular remodeling, endothelial dysfunction, and inflammation independently of hemodynamic effects [2,42]. Population-based data from the CARTaGENE cohort confirm that subclinical renin-independent aldosteronism, below conventional PA thresholds, associates with adverse cardiac remodeling and arterial stiffness, suggesting that earlier intervention may prevent progression to overt disease [13,43].

Expanding PA diagnosis has profound therapeutic implications. The predominantly intermediate lateralization probability among newly diagnosed patients (91% in ES 2025 patients vs 55% in ES 2016 patients), combined with their milder biochemical expression suggests that most are unlikely to benefit from adrenalectomy [44]. Per ES 2025 recommendations, these patients are instead candidates for shared decision making between empiric MRA therapy and further lateralization work-up, with most representing optimal candidates for early, mechanism-based MRA therapy with renin-guided titration [1,14,15,45].

Cardiovascular outcome evidence supporting early PA detection continues to accumulate. Cohort studies consistently demonstrate reduced cardiovascular events with appropriate PA therapy compared with conventional antihypertensive treatment at equivalent BP control with evidence that renin de-suppression optimizes cardiovascular outcomes even in milder phenotypes [[46], [47], [48]].

This pathophysiology-based management paradigm may help narrow the cardiovascular outcome gap between surgical and medical treatment [49]. Meta-analyses confirm that both adrenalectomy and MRA therapy reduce major adverse cardiovascular events, with surgical intervention demonstrating superior outcomes for unilateral disease [50,51]. Clinical prediction scoring supports individualized rather than systematic consideration for adrenalectomy, with AVS reserved for appropriate risk profiles [52,53].

**Limitations and future directions**

This study's single-center design with consecutive patient inclusion ensures homogeneous diagnostic practices and standardized protocols, while minimizing selection bias, and systematic documentation of missing data enhances transparency.

However, several limitations merit consideration. First, our cohort consists of patients referred by cardiologists for secondary HTN work-up and does not represent an unselected hypertensive population; PA prevalence is therefore enriched relative to primary care settings. Referral patterns may additionally have been influenced by growing awareness of the 2024 ESC and forthcoming ES 2025 recommendations during the study period, potentially contributing to broader referral indications. Our study evaluates diagnostic algorithm performance within this specialized referral setting and does not address population-level screening coverage, physician adherence, or the incremental benefit of extending systematic PA screening to primary care, which would require a separate prospective implementation study design. Second, laboratory-specific threshold requirements limit direct applicability of our specific cutpoints to other clinical settings, emphasizing the need for local validation efforts [1,[23], [24], [25]]. The local ARR threshold used for ES 2025 was derived from platform-specific validation against LC-MS/MS reference values rather than from a direct mathematical derivation. However, the same ARR screening criterion was applied in both algorithms; residual threshold effects therefore cannot account for differential reclassification between algorithms. Third, although the comparison of excluded (n = 29) versus included (n = 137) patients showed no statistically significant differences except for age (Supplementary Table S2), the numerically elevated proportions of severe HTN and obesity in excluded patients cannot be formally excluded as a source of non-random missingness. If severe phenotypes are systematically under-represented among included patients, true PA prevalence in the full population may be modestly higher than our primary analysis estimates. Sensitivity analyses addressing this scenario (all excluded patients PA-positive) yields a prevalence of 30.7% under ES 2025, demonstrating that even under maximal non-random assumptions, the directional finding is preserved. Fourth, adrenal CT is only indicated in patients with biochemically confirmed PA as part of the subtype classification work-up; CT does not establish the PA diagnosis, which is based on biochemical criteria alone, and CT data are therefore not available for the full screening cohort. Among patients who underwent CT at the time of data collection, imaging findings did not differ significantly across diagnostic groups (Supplementary Table S8). Fifth, the retrospective cross-sectional design precludes assessment of long-term cardiovascular outcomes and therapeutic responses, as MRA treatment response data fell outside of the scope of collected data. Sample size limitations affect certain subgroup analyses, particularly multivariable modeling, and the high NRI values should be interpreted with caution, as category-based NRI can yield large magnitudes in modest sample sizes. Finally, using ES 2025 criteria as the reference standard, while representing current evidence-based practice, acknowledges the inherent uncertainty in defining diagnostic truth for a condition without a perfect gold standard [54].

Critical research priorities include prospective multicenter validation that patients newly diagnosed by ES 2025 criteria experience improved cardiovascular outcomes with targeted therapy and optimization of MRA dosing strategies, and novel therapeutic agents for the milder phenotypes identified by expanded diagnosis. Diagnostic algorithm refinement through artificial intelligence and non-invasive lateralization functional imaging represent additional directions that could enhance diagnostic precision while reducing procedural requirements [55].

## 5 Conclusion

ES 2025 criteria nearly double PA detection by identifying milder phenotypes characterized by lower aldosterone levels, negative confirmatory tests, and intermediate lateralization probability, features suggesting limited benefit from adrenal vein sampling and surgical intervention. The high SIT false-negative rate supports abandoning mandatory confirmatory testing in favor of diagnosis based on the biochemical triad of suppressed renin, elevated aldosterone, and pathological ARR. Successful implementation requires laboratory-specific threshold validation. Prospective studies are now required to determine whether earlier identification and renin-guided MRA therapy in these newly diagnosed patients translate into improved long-term cardiovascular outcomes.

## CRediT authorship contribution statement

**Clara Cherdo:** Writing – original draft, Visualization, Validation, Investigation, Formal analysis, Data curation, Conceptualization. **Corina Mirea:** Writing – original draft, Visualization, Validation, Investigation. **Camille Zamperini:** Writing – original draft, Visualization, Validation, Investigation. **Solène Brouder:** Writing – original draft, Visualization, Validation, Investigation. **Dominique Stephan:** Writing – review & editing, Writing – original draft, Visualization, Validation, Supervision, Project administration, Methodology, Conceptualization. **Elena-Mihaela Cordeanu:** Writing – review & editing, Writing – original draft, Visualization, Validation, Supervision, Project administration, Methodology, Formal analysis, Data curation, Conceptualization.

## Declaration of generative AI and AI-assisted technologies in the writing process

During the preparation of this work the authors used CLAUDE AI and Chat GPT in order to improve language and readability of the manuscript. After using this tool, the authors reviewed and edited the content as needed and take full responsibility for the content of the publication.

## Funding

This research did not receive any specific grant from funding agencies in the public, commercial, or not-for-profit sectors.

## Declaration of competing interest

The authors declare no competing interests related to this work.

## Appendix A Supplementary data

Multimedia component 7 Multimedia component 7

Fig. S1 **Venn diagram showing diagnostic overlap between PA detection strategies** ES: Endocrine Society guidelines; PA: primary aldosteronism; SIT: saline infusion test. Fig. S1

## References

[1] Funder J.W., Carey R.M., Mantero F., Murad M.H., Reincke M., Shibata H. (2016). The management of primary aldosteronism: case detection, diagnosis, and treatment: an endocrine society clinical practice guideline. J. Clin. Endocrinol. Metab..

[2] Monticone S., Burrello J., Tizzani D., Bertello C., Viola A., Buffolo F. (2017). Prevalence and clinical manifestations of primary aldosteronism encountered in primary care practice. J. Am. Coll. Cardiol..

[3] Huang M., Li J., Zhao X., Fu R., Li X., Jiang W. (2024). Global and regional prevalence and cardiovascular risk of primary aldosteronism: a systematic review and meta-analysis. Curr. Probl. Cardiol..

[4] Hundemer G.L., Curhan G.C., Yozamp N., Wang M., Vaidya A. (2018). Renal outcomes in medically and surgically treated primary aldosteronism. Hypertens Dallas Tex 1979.

[5] Milliez P., Girerd X., Plouin P.-F., Blacher J., Safar M.E., Mourad J.-J. (2005). Evidence for an increased rate of cardiovascular events in patients with primary aldosteronism. J. Am. Coll. Cardiol..

[6] Monticone S., D'Ascenzo F., Moretti C., Williams T.A., Veglio F., Gaita F. (2018). Cardiovascular events and target organ damage in primary aldosteronism compared with essential hypertension: a systematic review and meta-analysis. Lancet Diabetes Endocrinol..

[7] Zhang H., Lin Z., Tian W., Ma Z. (2026). Genetic evidence for the causal relationship between metabolic diseases and primary aldosteronism: insights from Mendelian randomization analysis. Cardiovasc. Endocrinol. Metab..

[8] Inoue K., Naito T., Fuji R., Sonehara K., Yamamoto K., Baba R. (2024). Primary aldosteronism and risk of cardiovascular outcomes: genome-wide association and Mendelian randomization study. J. Am. Heart Assoc..

[9] Conn J.W. (1955). Presidential address. I. Painting background. II. Primary aldosteronism, a new clinical syndrome. J. Lab. Clin. Med..

[10] Papadopoulou-Marketou N., Vaidya A., Dluhy R., Chrousos G.P., Feingold K.R., Adler R.A., Ahmed S.F., Anawalt B., Blackman M.R., Chrousos G. (2000). Endotext.

[11] Vaidya A., Mulatero P., Baudrand R., Adler G.K. (2018). The expanding spectrum of primary aldosteronism: implications for diagnosis, pathogenesis, and treatment. Endocr. Rev..

[12] Brown J.M., Robinson-Cohen C., Luque-Fernandez M.A., Allison M.A., Baudrand R., Ix J.H. (2017). The spectrum of subclinical primary aldosteronism and incident hypertension: a cohort study. Ann. Intern. Med..

[13] Hundemer G.L., Agharazii M., Madore F., Vaidya A., Brown J.M., Leung A.A. (2024). Subclinical primary aldosteronism and cardiovascular health: a population-based cohort study. Circulation.

[14] Hundemer G.L., Curhan G.C., Yozamp N., Wang M., Vaidya A. (2018). Cardiometabolic outcomes and mortality in medically treated primary aldosteronism: a retrospective cohort study. Lancet Diabetes Endocrinol..

[15] Adler G.K., Stowasser M., Correa R.R., Khan N., Kline G., McGowan M.J. (2025). Primary aldosteronism: an endocrine society clinical practice guideline. J. Clin. Endocrinol. Metab..

[16] Monticone S., Goi J., Burrello J., Di Dalmazi G., Cicero A.F.G., Mancusi C. (2025). Screening of primary aldosteronism and pheochromocytoma among patients with hypertension: an Italian nationwide survey. J. Endocrinol. Investig..

[17] Schirpenbach C., Seiler L., Maser-Gluth C., Rüdiger F., Nickel C., Beuschlein F. (2006). Confirmatory testing in normokalaemic primary aldosteronism: the value of the saline infusion test and urinary aldosterone metabolites. Eur. J. Endocrinol..

[18] Betz M.J., Degenhart C., Fischer E., Pallauf A., Brand V., Bidlingmaier M. (2020). Confirmatory testing of primary aldosteronism with saline infusion test and LC-MS/MS. Endocr. Connect.

[19] Meng X., Li Y., Wang X., Li J., Liu Y., Yu Y. (2018). Evaluation of the saline infusion test and the captopril challenge test in Chinese patients with primary aldosteronism. J. Clin. Endocrinol. Metab..

[20] Golani T., Bleier J., Kaplan A., Hod T., Sharabi Y., Leibowitz A. (2024). A 120-Minute saline infusion test for the confirmation of primary aldosteronism: a pilot study. Am. J. Hypertens..

[21] Gunnarsdottir H., Jonsdottir G., Birgisson G., Gudmundsson J., Sigurjonsdottir H.A. (2022). Are we only detecting the tip of the iceberg? A nationwide study on primary aldosteronism with up to 8-Year Follow-up. Endocr. Res..

[22] ESH. Directory of Excellence Centres n.d. https://www.eshonline.org/communities/excellence-centres/directory-of-excellence-centres/?country=France (accessed January 6, 2026).

[23] Fischer E., Reuschl S., Quinkler M., Rump L., Hahner S., Bidlingmaier M. (2013). Assay characteristics influence the aldosterone to renin ratio as a screening tool for primary aldosteronism: results of the German conn's registry. Horm. Metab. Res..

[24] Williams T.A., Reincke M. (2018). Management of endocrine disease: diagnosis and management of primary aldosteronism: the endocrine society guideline 2016 revisited. Eur. J. Endocrinol..

[25] Denimal D., Duvillard L. (2016). 2016 endocrine society guidelines update for the diagnosis of primary aldosteronism: are the proposed aldosterone-to-renin ratio cut-off values relevant in the era of fully automated immunoassays?. Ann. Clin. Biochem..

[26] Manolopoulou J., Fischer E., Dietz A., Diederich S., Holmes D., Junnila R. (2015). Clinical validation for the aldosterone-to-renin ratio and aldosterone suppression testing using simultaneous fully automated chemiluminescence immunoassays. J. Hypertens..

[27] Vittinghoff E., McCulloch C.E. (2007). Relaxing the rule of ten events per variable in logistic and cox regression. Am. J. Epidemiol..

[28] Xu Z., Yang J., Hu J., Song Y., He W., Luo T. (2020). Primary aldosteronism in patients in China with recently detected hypertension. J. Am. Coll. Cardiol..

[29] Libianto R., Russell G.M., Stowasser M., Gwini S.M., Nuttall P., Shen J. (2022). Detecting primary aldosteronism in Australian primary care: a prospective study. Med. J. Aust..

[30] Wada N., Miyoshi A., Usubuchi H., Terae S., Shibayama Y., Takahashi B. (2021). Prediction of unilateral hyperaldosteronism on adrenal vein sampling using captopril challenge test in patients with primary aldosteronism. Endocr. J..

[31] Buffolo F., Pecori A., Reincke M., Outland M., Veglio F., Schwarzlmüller P. (2024). Long-term follow-up of patients with elevated aldosterone-to-renin ratio but negative confirmatory test: the progression of primary aldosteronism phenotypes. Hypertension.

[32] Leung A.A., Symonds C.J., Hundemer G.L., Ronksley P.E., Lorenzetti D.L., Pasieka J.L. (2022). Performance of confirmatory tests for diagnosing primary aldosteronism: a systematic review and meta-analysis. Hypertension.

[33] Zhu R., Shagjaa T., Rossitto G., Caroccia B., Seccia T.M., Gregori D. (2023). Exclusion tests in unilateral primary aldosteronism (ExcluPA) study. J. Clin. Endocrinol. Metab..

[34] McEvoy J.W., McCarthy C.P., Bruno R.M., Brouwers S., Canavan M.D., Ceconi C. (2024). 2024 ESC guidelines for the management of elevated blood pressure and hypertension. Eur. Heart J..

[35] Baron S., Amar L., Faucon A.L., Blanchard A., Baffalie L., Faucard C. (2018). Criteria for diagnosing primary aldosteronism on the basis of liquid chromatography-tandem mass spectrometry determinations of plasma aldosterone concentration. J. Hypertens..

[36] Douillard C., Houillier P., Nussberger J., Girerd X. (2016). SFE/SFHTA/AFCE consensus on primary aldosteronism, part 2: first diagnostic steps. Ann. Endocrinol..

[37] Sealey J.E. (1991). Plasma renin activity and plasma prorenin assays. Clin. Chem..

[38] Stowasser M., Ahmed A.H., Pimenta E., Taylor P.J., Gordon R.D. (2012). Factors affecting the aldosterone/renin ratio. Horm. Metab. Res..

[39] Ito S., Itoh H., Rakugi H., Okuda Y., Yoshimura M., Yamakawa S. (2020). Double-blind randomized phase 3 study comparing esaxerenone (CS-3150) and eplerenone in patients with essential hypertension (ESAX-HTN study). Hypertension.

[40] Cicero A.F.G., Tocci G., Avagimyan A., Penson P., Nardoianni G., Perone F. (2025). Aldosterone synthase inhibitors for resistant hypertension: pharmacological insights - a systematic review. Drugs.

[41] Ho W.Y., Hsiao C.C., Wu P.H., Chen J.Y., Tu Y.K., Wu V.C. (2024). Comparison of different medical treatments for primary hyperaldosteronism: a systematic review and network meta-analysis. Ther. Adv. Chronic Dis..

[42] Parasiliti-Caprino M., Lopez C., Prencipe N., Lucatello B., Settanni F., Giraudo G. (2020). Prevalence of primary aldosteronism and association with cardiovascular complications in patients with resistant and refractory hypertension. J. Hypertens..

[43] Goupil R., Desbiens L.-C., Merabtine A., Agharazii M., Madore F., Vaidya A. (2025). Subclinical primary aldosteronism and major adverse cardiovascular events: a longitudinal population-based cohort study. Circulation.

[44] Young W.F. (2019). Diagnosis and treatment of primary aldosteronism: practical clinical perspectives. J. Intern. Med..

[45] Farrugia F.A., Zavras N., Martikos G., Tzanetis P., Charalampopoulos A., Misiakos E.P. (2018). A short review of primary aldosteronism in a question and answer fashion. Endocr. Regul..

[46] Huang C.W., Huang T.Y., Yang Y.F., Chang L.Y., Tu Y.K., Wu V.C. (2025). Major adverse cardiovascular events in primary aldosteronism after adrenalectomy or mineralocorticoid receptor antagonist treatment: a systematic review and meta-analysis. J. Am. Heart Assoc..

[47] Chen S.Y., Chen J.Y., Huang W.C., Puar T.H.K., Chin Kek P., Chueh J.S. (2022). Cardiovascular outcomes and all-cause mortality in primary aldosteronism after adrenalectomy or mineralocorticoid receptor antagonist treatment: a meta-analysis. Eur. J. Endocrinol..

[48] Hundemer G.L., Leung A.A., Kline G.A., Brown J.M., Turcu A.F., Vaidya A. (2024). Biomarkers to guide medical therapy in primary aldosteronism. Endocr. Rev..

[49] Jing Y., Liao K., Li R., Zhang X., Liu J., Chen J. (2022). Cardiovascular events and all-cause mortality in surgically or medically treated primary aldosteronism: a meta-analysis. J. Renin-Angiotensin-Aldosterone Syst. JRAAS.

[50] Nayak S.S., Amini-Salehi E., Joukar F., Biswas P., Nobakht S., Letafatkar N. (2024). Cardiovascular and all-cause mortality outcomes of adrenalectomy versus medical treatment in primary aldosteronism: an umbrella review. Int. J. Surg..

[51] Wolley M.J., Gordon R.D., Ahmed A.H., Stowasser M. (2015). Does contralateral suppression at adrenal venous sampling predict outcome following unilateral adrenalectomy for primary aldosteronism? A retrospective study. J. Clin. Endocrinol. Metab..

[52] Küpers E.M., Amar L., Raynaud A., Plouin P.F., Steichen O. (2012). A clinical prediction score to diagnose unilateral primary aldosteronism. J. Clin. Endocrinol. Metab..

[53] Marzano L., Husain-Syed F., Reis T., Ronco C., Zanella M. (2023). Assessment of performance of stratum-specific likelihood ratios of the aldosteronoma resolution score for predicting hypertension cure after adrenalectomy for primary aldosteronism: a systematic review and meta-analysis. J. Hum. Hypertens..

[54] Funder J.W., Carey R.M. (2022). Primary aldosteronism: where are we now? Where to from here?. Hypertension.

[55] de Freminville J.B., Gardini M., Cremer A., Camelli S., Baron S., Bobrie G. (2024). Prevalence and risk factors for secondary hypertension in young adults. Hypertension.

