# Supplementary material for: Endocrine society 2025 diagnostic criteria increase primary aldosteronism detection in hypertensive patients: a comparative study with 2016 guidelines
Source: Int J Cardiol Cardiovasc Risk Prev. 2026 Apr 12;29:200638. doi: 10.1016/j.ijcrp.2026.200638 (PMC13096894; doi:10.1016/j.ijcrp.2026.200638)
Supplement: Multimedia component 1 [file mmc1.docx]

**Supplementary Table S7. Multivariate analysis of factors associated with PA diagnosis according to ES 2025 criteria**

| **Variable** | **Univariate OR [95% CI]** | **p-value** | **Adjusted OR [95% CI]** | **p-value** |
| --- | --- | --- | --- | --- |
| Resistant hypertension | 2.22 [0.82-6.02] | 0.15 | 1.95 [0.68-5.62] | 0.21 |
| Hypokalemia (≤3.5 mmol/L) | 2.22 [0.82-6.02] | 0.15 | 2.05 [0.71-5.89] | 0.18 |
| **Positive SIT (>50 ng/L)** | **3.71 [1.44-9.57]** | **0.008*** | **3.25 [1.22-8.67]** | **0.019*** |

CI: confidence interval; OR: odds ratio; PA: primary aldosteronism; SIT: saline infusion test. Model performance: AUC = 0.72, Hosmer-Lemeshow p = 0.42. *p<0.05
