# Supplementary material for: Endocrine society 2025 diagnostic criteria increase primary aldosteronism detection in hypertensive patients: a comparative study with 2016 guidelines
Source: Int J Cardiol Cardiovasc Risk Prev. 2026 Apr 12;29:200638. doi: 10.1016/j.ijcrp.2026.200638 (PMC13096894; doi:10.1016/j.ijcrp.2026.200638)
Supplement: Multimedia component 2 [file mmc2.docx]

**Supplementary Table S1. Hormonal parameters across diagnostic groups**

| **Parameter** | **Total (n=137)** | **ES 2016 (n=12)** | **ES 2025 (n=22)** | **No PA (n=114)** | **p-value*** |
| --- | --- | --- | --- | --- | --- |
| ***Baseline measurements*** | | | | | |
| Aldosterone. ng/L. median [IQR] | 87.5 [48.9-151] | 167.5 [126-202] | 120.5 [90.9-169] | 72.0 [37-114] | **<0.001** |
| Renin. ng/L. median [IQR] | 8.9 [3.4-17.0] | 1.9 [1.1-3.4] | 2.1 [1.4-2.5] | 12.2 [7.0-21.9] | **<0.001** |
| Pathological ARR (>18.7). n (%) | 40 (29.2) | 12 (100) | 22 (100) | 17 (14.9) | **<0.001** |
| ***Post-saline infusion test*** | | | | | |
| Aldosterone. ng/L. median [IQR] | 37.0 [37-68.4] | 71.7 [53.8-99.7] | 56.2 [40.7-80.7] | 37.0 [37-49.4] | **<0.001** |
| Renin. ng/L. median [IQR] | 5.9 [2.3-12.2] | 1.5 [1.1-2.1] | 1.8 [1.1-2.3] | 8.5 [4.4-14.7] | **<0.001** |
| Positive SIT (>50 ng/L). n (%) | 40 (29.2) | 10 (83.3) | 14 (63.6) | 26 (22.8) | **<0.001** |
| Positive SIT (>100 ng/L). n (%) | 12 (8.8) | 6 (50.0) | 3 (13.6) | 9 (7.9) | **<0.001** |

ARR: aldosterone-to-renin ratio; ES: Endocrine Society; IQR: interquartile range; PA: primary aldosteronism; SIT: saline infusion test. *p-values represent comparisons between ES 2025 and No PA groups.
