# Supplementary material for: Endocrine society 2025 diagnostic criteria increase primary aldosteronism detection in hypertensive patients: a comparative study with 2016 guidelines
Source: Int J Cardiol Cardiovasc Risk Prev. 2026 Apr 12;29:200638. doi: 10.1016/j.ijcrp.2026.200638 (PMC13096894; doi:10.1016/j.ijcrp.2026.200638)
Supplement: Multimedia component 3 [file mmc3.docx]

**Supplementary Table S2. Baseline characteristics of excluded versus included patients**

| **Characteristic** | **Excluded (n=29)** | **Included (n=137)** | **p-value** |
| --- | --- | --- | --- |
| ***Demographics*** | | | |
| Age, years, median [IQR] | 52 [42-65] | 41 [34-53] | **<0.01** |
| Male sex, n (%) | 13 (44.8) | 73 (53.3) | 0.53 |
| BMI, kg/m², mean ± SD | 32.5 ± 9.5* | 27.8 ± 5.2 | 0.19 |
| ***Hypertension characteristics*** | | | |
| Recent onset (<1 year), n (%) | 7 (63.6)* | 66 (48.2) | 0.36 |
| Severe hypertension, n (%) | 6 (54.5)* | 54 (39.4) | 0.35 |
| Resistant hypertension, n (%) | 1 (9.1)* | 27 (19.7) | 0.69 |
| Office SBP, mmHg, median [IQR] | 148 [144-173]* | 150 [136-169] | 0.78 |
| Office DBP, mmHg, median [IQR] | 92 [88-96]* | 89 [80-97] | 0.55 |
| ***Laboratory findings*** | | | |
| Hypokalemia (≤3.5 mmol/L), n (%) | 4 (33.3)* | 27 (19.7) | 0.27 |
| eGFR, mL/min/1.73m², median [IQR] | 108 [100-115]* | 104 [90-115] | 0.18 |
| ***Reason for exclusion*** | | | |
| Technical assay failure, n (%) | 18 (62.1) | — | — |
| Sample collection issues, n (%) | 11 (37.9) | — | — |

BMI: body mass index; DBP: diastolic blood pressure; eGFR: estimated glomerular filtration rate; IQR: interquartile range; SBP: systolic blood pressure; SD: standard deviation. *Data available for a subset of excluded patients (n=11-12). Comparisons used Mann-Whitney U test for continuous variables and Fisher’s exact test for categorical variables.
