# Supplementary material for: Endocrine society 2025 diagnostic criteria increase primary aldosteronism detection in hypertensive patients: a comparative study with 2016 guidelines
Source: Int J Cardiol Cardiovasc Risk Prev. 2026 Apr 12;29:200638. doi: 10.1016/j.ijcrp.2026.200638 (PMC13096894; doi:10.1016/j.ijcrp.2026.200638)
Supplement: Multimedia component 4 [file mmc4.docx]

**Supplementary Table S3. Sensitivity analysis for missing data**

| **Scenario** | **ES 2016 prevalence** | **ES 2025 prevalence** |
| --- | --- | --- |
| Primary analysis (n=137) | 8.8% (12/137) | 16.1% (22/137) |
| Best-case (all excluded PA+) | 24.7% (41/166) | 30.7% (51/166) |
| Worst-case (all excluded PA−) | 7.2% (12/166) | 13.3% (22/166) |

ES: Endocrine Society; PA+ : primary aldosteronism positive; PA− : primary aldosteronism negative. Best-case scenario assumes all 29 excluded patients would have met diagnostic criteria. Worst-case scenario assumes none of the excluded patients would have met diagnostic criteria.
