# Supplementary material for: Endocrine society 2025 diagnostic criteria increase primary aldosteronism detection in hypertensive patients: a comparative study with 2016 guidelines
Source: Int J Cardiol Cardiovasc Risk Prev. 2026 Apr 12;29:200638. doi: 10.1016/j.ijcrp.2026.200638 (PMC13096894; doi:10.1016/j.ijcrp.2026.200638)
Supplement: Multimedia component 5 [file mmc5.docx]

**Supplementary Table S4. Inter-method agreement analysis**

| **Comparison** | **Observed Agreement (Po)** | **Expected Agreement (Pe)** | **Kappa (κ)** | **95% CI** | **Interpretation** |
| --- | --- | --- | --- | --- | --- |
| **ES 2025 vs ES 2016 permissive** | 91.2% | 77.4% | **0.61** | [0.42 – 0.80] | Substantial |
| **ES 2025 vs ES 2016 restrictive** | 86.9% | 82.1% | **0.27** | [0.04 – 0.50] | Fair |
| **ES 2025 vs SIT permissive** | 75.2% | 56.8% | **0.43** | [0.28 – 0.58] | Moderate |
| **ES 2025 vs SIT restrictive** | 79.6% | 77.9% | **0.08** | [-0.08 – 0.24] | Slight |
| **ES 2016 permissive vs ES 2016 restrictive** | 95.6% | 91.5% | **0.48** | [0.24 – 0.72] | Moderate |
| **SIT permissive vs SIT restrictive** | 78.8% | 74.4% | **0.17** | [0.03 – 0.31] | Fair |

CI: Confidence Interval; ES: Endocrine Society; SIT: Saline Infusion Test (permissive: >50 ng/L; restrictive: >100 ng/L). Kappa formula: κ = (Po – Pe) / (1 – Pe).
