# Supplementary material for: Endocrine society 2025 diagnostic criteria increase primary aldosteronism detection in hypertensive patients: a comparative study with 2016 guidelines
Source: Int J Cardiol Cardiovasc Risk Prev. 2026 Apr 12;29:200638. doi: 10.1016/j.ijcrp.2026.200638 (PMC13096894; doi:10.1016/j.ijcrp.2026.200638)
Supplement: Multimedia component 6 [file mmc6.docx]

**Supplementary Table S5. Diagnostic performance of alternative algorithms vs ES 2025**

| **Diagnostic strategy** | **PA**  **n (%)** | **Sensitivity** | **Specificity** | **PPV** | **NPV** |
| --- | --- | --- | --- | --- | --- |
| **ES 2025 (Reference)** | 22 (16.1) | — | — | — | — |
| **ES 2016 permissive** | 12 (8.8) | 0.50 | 0.99 | 0.92 | 0.91 |
| **ES 2016 restrictive** | 6 (4.4) | 0.23 | 0.99 | 0.83 | 0.87 |
| **SIT alone (permissive)** | 40 (29.2) | 0.64 | 0.77 | 0.35 | 0.92 |
| **SIT alone (restrictive)** | 12 (8.8) | 0.14 | 0.92 | 0.25 | 0.85 |

CI: confidence interval; ES: Endocrine Society; n: number; NPV: negative predictive value; PA: primary aldosteronism; PPV: positive predictive value; SIT: saline infusion test.
