# Supplementary material for: Endocrine society 2025 diagnostic criteria increase primary aldosteronism detection in hypertensive patients: a comparative study with 2016 guidelines
Source: Int J Cardiol Cardiovasc Risk Prev. 2026 Apr 12;29:200638. doi: 10.1016/j.ijcrp.2026.200638 (PMC13096894; doi:10.1016/j.ijcrp.2026.200638)
Supplement: Multimedia component 7 [file mmc7.docx]

**Supplementary Table S6. Net Reclassification Improvement (NRI) analysis**

| **Comparison** | **Event NRI (%)** | **Non-event NRI (%)** | **Overall NRI (%)** | **p-value** |
| --- | --- | --- | --- | --- |
| ES 2025 vs ES 2016 (permissive) | +50 | +98 | +148 | **0.009** |
| ES 2025 vs ES 2016 (restrictive) | +77 | +98 | +175 | **<0.001** |
| ES 2025 vs SIT alone (permissive) | +36 | +55 | +91 | **0.004** |
| ES 2025 vs SIT alone (restrictive) | -22 | +92 | +70 | **0.02** |

ES: Endocrine Society; NRI: net reclassification improvement; SIT: saline infusion test. Event NRI reflects improvement in classification of true PA cases; Non-event NRI reflects improvement in classification of true non-PA cases.
