# Supplementary figures and images for: Endocrine society 2025 diagnostic criteria increase primary aldosteronism detection in hypertensive patients: a comparative study with 2016 guidelines
Source: Int J Cardiol Cardiovasc Risk Prev. 2026 Apr 12;29:200638. doi: 10.1016/j.ijcrp.2026.200638 (PMC13096894; doi:10.1016/j.ijcrp.2026.200638)

**Total: 137 patients**

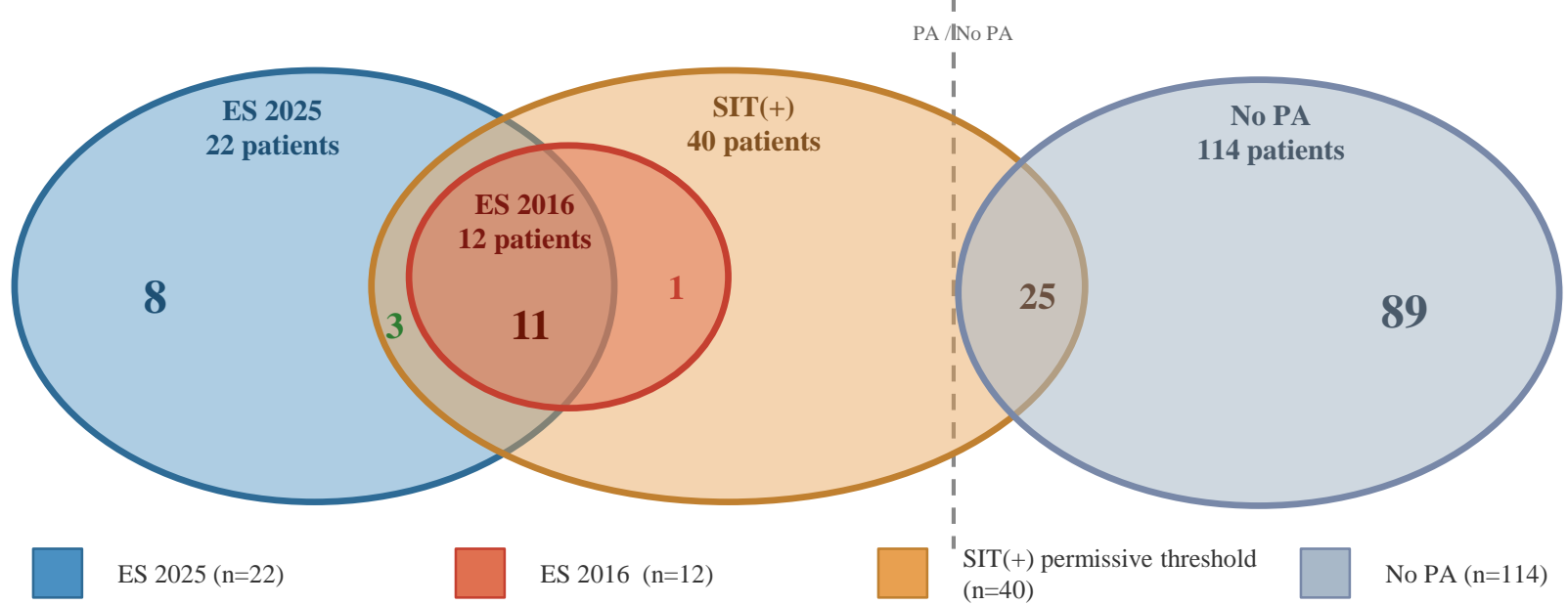

Supplement: Fig. S1 — Venn diagram showing diagnostic overlap between PA detection strategies ES: Endocrine Society guidelines; PA: primary aldosteronism; SIT: saline infusion test. [file mmc8.pdf]
