# Supplementary material for: Endocrine society 2025 diagnostic criteria increase primary aldosteronism detection in hypertensive patients: a comparative study with 2016 guidelines
Source: Int J Cardiol Cardiovasc Risk Prev. 2026 Apr 12;29:200638. doi: 10.1016/j.ijcrp.2026.200638 (PMC13096894; doi:10.1016/j.ijcrp.2026.200638)
Supplement: Multimedia component 9 [file mmc9.docx]

# Supplementary Methods

# Laboratory assessment protocols

# Primary Aldosteronism (PA)-specific testing protocols

All laboratory specimens were collected under standardized conditions to minimize pre-analytical variability:

- Morning collection between 8:00-9:00 AM after overnight fasting when required
- Immediate processing or appropriate storage conditions for delayed analysis

## Sample collection standardization for hormonal assessment

### Postural standardization:

- Patients ambulatory for minimum 2 hours before sampling to ensure renin activation
- Seated position maintained for 15 minutes immediately prior to phlebotomy
- Avoidance of supine positioning which suppresses renin and elevates aldosterone

### Dietary considerations:

- Normal dietary sodium intake maintained for 3 days prior to testing
- Potassium supplementation to correct hypokalemia when present before testing

### *Medication management:*

Systematic approach to medication modification when clinically safe and feasible:

- ACE inhibitors and ARBs discontinued 2 weeks prior to testing when possible
- Beta-blockers, Thiazide, loop diuretics and potassium-sparing diuretics discontinued 4 weeks prior due to prolonged renin suppression effects
- Calcium channel blockers and alpha-blockers or centrally acting drugs were continued as minimally interfering
- Documentation of medications unable to discontinue for safety reasons

## Inter-laboratory comparison and threshold derivation

Following established recommendations emphasizing that diagnostic thresholds are assay-dependent and require local validation (Fischer et al. 2013; Denimal and Duvillard 2016; ES 2016; ES 2025), we adopted platform-specific cutoffs validated for the iSYS automated chemiluminescent immunoassay used at our institution, and performed inter-laboratory comparison studies to confirm the expected systematic differences in DRC measurements.

## Inter-laboratory comparison methodology

- Split-sample analysis was performed on 45 consecutive patient samples spanning the clinically relevant range of renin and aldosterone concentrations. Samples were simultaneously analyzed at our institution using the iSYS platform (IDS Corporation) and at two French reference laboratories using alternative immunoassay methods (DiaSorin LIAISON and Siemens IMMULITE). Results demonstrated systematic differences in direct renin concentration (DRC) measurements:
- iSYS DRC values were 24.7% higher (95% CI: 21.3-28.1%) compared to the DiaSorin LIAISON platform
- iSYS DRC values were 25.2% higher (95% CI: 22.0-28.4%) compared to the Siemens IMMULITE platform
- Mean correction factor: 1.25 (25% elevation)
- Correlation coefficient (r²) between platforms: 0.94 for DRC, 0.97 for aldosterone
- Aldosterone measurements showed good agreement across platforms (mean difference: 3.2%, within acceptable variation), therefore aldosterone thresholds were maintained as recommended by ES 2025 guidelines.

## Threshold derivation:

### Aldosterone-to-Renin Ratio (ARR) threshold:

Our ARR threshold of >18.7 (aldosterone ng/L / DRC ng/L) was adopted from the iSYS-platform-specific validation by Manolopoulou et al. (2015), who established this cutoff in 152 patients with 99% sensitivity and 79% specificity for PA diagnosis using the same automated chemiluminescent immunoassay (iSYS, IDS Corporation) used at our institution.

The ES 2025 guidelines provide ARR cutoffs in multiple unit combinations. For immunoassay-measured aldosterone with DRC: ARR >70 (pmol/L per mU/L), equivalent to >18.75 (ng/L per mIU/L). For LC-MS/MS-measured aldosterone with DRC: ARR >52 (pmol/L per mIU/L), approximately 25% lower. Our iSYS aldosterone measurements correlate well with LC-MS/MS (mean difference 3.2%); therefore, our Manolopoulou-derived threshold of >18.7 (ng/ng) equivalent to >52 (pmol/ng) and >11.2 (ng/mIU), aligns with the LC-MS/MS-based cutoffs.

### Direct Renin Concentration (DRC) threshold:

ES 2025 recommended threshold for suppressed renin: DRC ≤8.2 mIU/L

Our inter-laboratory comparison demonstrated that the iSYS platform yields DRC values 25% higher than reference laboratories (correction factor: 1.25). Adjustment: 8.2 × 1.25 = 10.25 mIU/L. Conversion from mIU/L to ng/L (conversion factor: 1.667): 10.25 ÷ 1.667 = 6.148 ng/L

Local threshold applied: DRC ≤6.15 ng/L (equivalent to ≤10.25 mIU/L)

### Aldosterone threshold:

ES 2025 recommended thresholds: aldosterone ≥100 ng/L (10 ng/dL) by immunoassay; ≥75 ng/L (7.5 ng/dL) by LC-MS/MS. Inter-laboratory comparison showed minimal systematic difference in aldosterone between iSYS and LC-MS/MS reference methods (mean difference: 3.2%), in contrast to the significant 25% bias observed for DRC. Therefore, the LC-MS/MS aldosterone threshold applies to our platform. Local threshold applied: aldosterone ≥75 ng/L (corresponding to LC-MS/MS threshold of ≥7.5 ng/dL)

## Hormone measurement technical specifications

### Aldosterone measurement (iSYS Platform, IDS Corporation):

- Automated chemiluminescent immunoassay with monoclonal antibodies
- Measurement range: 10-1000 ng/L with appropriate dilution for higher values
- Intra-assay coefficient of variation: <8% at clinically relevant concentrations
- Inter-assay coefficient of variation: <12% based on quality control data
- Cross-reactivity: <2% with other mineralocorticoids and corticosteroids
- Analytical sensitivity: 5 ng/L based on manufacturer specifications
- Reference intervals established through local healthy population studies

### Direct Renin Concentration (DRC) measurement (iSYS Platform):

- Automated immunoassay measuring active renin concentration only
- Measurement range: 1-100 ng/L with appropriate sample dilution when needed
- Intra-assay coefficient of variation: <10% at normal and elevated concentrations
- Inter-assay coefficient of variation: <15% based on internal quality control
- Temperature sensitivity requiring careful pre-analytical sample handling
- No cross-reactivity with inactive prorenin forms
- Local reference intervals adjusted for 25% higher values compared to other French laboratories

### Quality control and external validation:

- Daily internal quality control using manufacturer-provided standards
- Participation in external quality assessment programs for hormone measurements
- Inter-laboratory comparison studies to validate local threshold adjustments
- Correlation studies with LC-MS/MS reference methods when available
- Regular calibration verification and maintenance according to manufacturer protocols

## Saline Infusion Test (SIT) detailed protocol

### Infusion parameters and timeline:

- Total volume: 2000 mL normal saline administered over exactly 4 hours
- Patient positioning: strict supine position maintained during entire infusion

### Monitoring protocol during infusion:

- Vital signs (blood pressure, heart rate, respiratory rate) every 30 minutes
- Clinical assessment for fluid overload signs every 30 minutes

**Post-infusion assessment and sample collection:** *immediate post-infusion procedures:*

- Sample collection within 15 minutes of infusion completion

# Statistical analysis detailed methodology

## Descriptive statistics and data presentation:

All baseline characteristics and outcome variables were analyzed using appropriate statistical methods based on data type and distribution characteristics:

### Continuous variables:

- Normality assessment using Shapiro-Wilk test with alpha = 0.05
- Normal distributions presented as mean ± standard deviation
- Non-normal distributions presented as median [interquartile range]
- Range and outlier assessment with appropriate data verification
- Missing data patterns analyzed and reported for all variables

### Categorical variables:

- Frequency distributions with percentages calculated for non-missing data
- 95% confidence intervals for proportions using exact binomial methods
- Cross-tabulation with relevant covariates when clinically appropriate
- Missing data explicitly reported for all categorical analyses

## Comparative analysis methodology:

### Two-group comparisons:

- Mann-Whitney U test for continuous variables with non-normal distributions
- Student's t-test for normally distributed continuous variables with equal variances
- Welch's t-test for normally distributed variables with unequal variances
- Chi-square test for categorical variables when expected cell counts ≥5
- Fisher's exact test for categorical variables with expected cell counts <5

### Multiple group comparisons:

- Kruskal-Wallis test for continuous variables across three diagnostic groups
- Chi-square test for categorical variables across multiple groups

# Diagnostic performance analysis methodology*:*

Using ES 2025 criteria as reference standard, comprehensive diagnostic performance metrics calculated for all alternative approaches:

- Sensitivity = True Positives / (True Positives + False Negatives)
- Specificity = True Negatives / (True Negatives + False Positives)
- Positive Predictive Value = True Positives / (True Positives + False Positives)
- Negative Predictive Value = True Negatives / (True Negatives + False Negatives)
- Diagnostic Accuracy = (True Positives + True Negatives) / Total Population

**Agreement analysis technical details:**

*Cohen's kappa coefficient:*

- Formula: κ = (Po - Pe) / (1 - Pe) where Po = observed agreement, Pe = expected agreement by chance
- Interpretation: κ < 0.20 (poor), 0.20-0.40 (fair), 0.40-0.60 (moderate), 0.60-0.80 (substantial), >0.80 (almost perfect)
- 95% confidence intervals calculated using standard error formula
- Statistical significance testing using z-test with null hypothesis κ = 0

*McNemar's test for paired comparisons:*

- Application to paired diagnostic data where same patients evaluated with different algorithms
- Test statistic calculation: χ² = (b - c)² / (b + c) where b and c are discordant pairs
- Continuity correction applied when total discordant pairs < 25
- Exact binomial test used when McNemar assumptions not met

**Net Reclassification Improvement analysis:** *NRI calculation methodology:*

- Formula: NRI = [P(up|D+) - P(down|D+)] + [P(down|D-) - P(up|D-)]
- Event NRI = improvement in classification of true positive cases
- Non-event NRI = improvement in classification of true negative cases
- Overall NRI = Event NRI + Non-event NRI
- 95% confidence intervals calculated using bootstrap methods with 1000 resamples

*Interpretation guidelines:*

- NRI > 0 indicates net improvement in classification
- Clinical significance thresholds: NRI > 10% modest improvement, NRI > 20% substantial improvement
- Separate analysis of event and non-event reclassification to understand improvement sources

**Multivariate analysis:** *variable selection criteria:*

- Clinical relevance based on established pathophysiology and previous literature
- Statistical screening using p < 0.20 threshold in univariate analysis
- Assessment of collinearity using correlation matrices and variance inflation factors
- Minimum events per variable ratio of 10:1 maintained to prevent overfitting

# Software and computational details:

- R Statistical Software Version 4.4.1 (R Foundation for Statistical Computing, Vienna, Austria)
- Relevant packages: 'epi' for diagnostic test evaluation, 'irr' for agreement analysis, 'rms' for regression modeling

# Supplementary Methods References

- Manolopoulou J, Fischer E, Dietz A, et al. Clinical validation for the aldosterone-to-renin ratio and aldosterone suppression testing using simultaneous fully automated chemiluminescence immunoassays. J Hypertens 2015;33:2500–2511.
- O’Shea PM, Griffin TP, Browne GA, et al. Screening for primary aldosteronism using the aldosterone/renin ratio: how to adjust for the confounding effect of blood sampling conditions. Steroids 2016;111:46–53.
- Fischer E, Reuschl S, Quinkler M, et al. Assay characteristics influence the aldosterone to renin ratio as a screening tool for primary aldosteronism: results of the German Conn’s registry. Horm Metab Res 2013;45:526–531.
- Denimal D, Duvillard L. 2016 Endocrine Society guidelines update for the diagnosis of primary aldosteronism: are the proposed aldosterone-to-renin ratio cut-off values relevant in the era of fully automated immunoassays? Ann Clin Biochem 2016;53:714–715.
- Baron S, Amar L, Faucon AL, et al. Criteria for diagnosing primary aldosteronism on the basis of liquid chromatography-tandem mass spectrometry determinations of plasma aldosterone concentration. J Hypertens 2018;36:1592–1601.
