# Supplementary material for: Endocrine society 2025 diagnostic criteria increase primary aldosteronism detection in hypertensive patients: a comparative study with 2016 guidelines
Source: Int J Cardiol Cardiovasc Risk Prev. 2026 Apr 12;29:200638. doi: 10.1016/j.ijcrp.2026.200638 (PMC13096894; doi:10.1016/j.ijcrp.2026.200638)
Supplement: Multimedia component 10 [file mmc10.docx]

**Supplementary Table S8. Adrenal CT findings in patients who underwent imaging**

| **CT finding** | **PA(+) / ES 2016 (n=6)** | **PA(+) / ES 2025 (n=8)** | **No PA**  **(n=22)** | **p-value** |
| --- | --- | --- | --- | --- |
| Normal | 2 (33.3%) | 2 (25.0%) | 4 (18.2%) | 0.153 |
| Unilateral hyperplasia | 1 (16.7%) | 3 (37.5%)‡ | 5 (22.7%) |  |
| Bilateral hyperplasia | 0 (0%) | 0 (0%) | 1 (4.5%) |  |
| Unilateral nodule | 1 (16.7%) | 1 (12.5%) | 12 (54.5%) |  |
| Bilateral nodules | 2 (33.3%) | 2 (25.0%) | 0 (0%) |  |

CT: computed tomography; ES: Endocrine Society; n: number; PA: primary aldosteronism.
